# Tamoxifen induces region-specific osteocytic recombination and transiently alters bone structure in Dmp1-Cre-ERT2 mice

**DOI:** 10.1016/j.jbc.2026.113275

**Published:** 2026-06-22

**Authors:** Natalie YY. Koh, Natalie KY. Wee, Narelle E. McGregor, Ingrid J. Poulton, Milana Kuropatkina, Christa Maes, Natalie A. Sims

**Affiliations:** 1 Bone Cell Biology and Disease Unit, St Vincent’s Institute of Medical Research, Fitzroy, Melbourne, Australia; 2 Department of Medicine at St Vincent’s Hospital, The University of Melbourne, Melbourne, Australia; 3 Laboratory of Skeletal Cell Biology and Physiology (SCEBP), Skeletal Biology and Engineering Research Center (SBE), Department of Development and Regeneration, KU Leuven, Leuven, Belgium; 4 Mary Mackillop Institute for Health Research, Australian Catholic University, Melbourne, Australia

**Keywords:** transgenic mice, osteocyte, osteoblast, bone, estrogen receptor, recombination, sex differences

## Abstract

Tamoxifen is widely used to activate Cre-ERT2/LoxP transgenic systems for cell- and time-specific gene recombination. However, its regional and long-term effects on the adult murine skeleton are not well-defined. Here, we sought to identify immediate and long-term regional effects of short-term tamoxifen treatment on gene recombination and on cortical and trabecular bone structure in young adult ^10kb^Dmp1-Cre-ERT2 mice, which targets gene recombination primarily to osteoblasts and osteocytes. Tamoxifen was administered by four 20 mg/kg bolus injections to 12-week-old mice of both sexes. In cortical bone, tamoxifen induced persistent recombination in osteocytes (detected by Ai9.tdTomato labeling), which was region- and sex-dependent: female metaphyseal cortex showed the highest labeling (in ∼80% of osteocytes) that remained for 12 weeks. However, diaphyseal cortex and males showed less osteocyte labeling (∼60%). In trabecular bone of male and female mice, labeling was observed in ∼90% of osteocytes and in ∼60% of bone surface cells at 14 weeks of age but both substantially declined after a further 12 weeks, particularly in females. In both sexes, tamoxifen transiently accelerated longitudinal bone growth, increased trabecular bone mass, and increased metaphyseal cortical porosity while suppressing radial growth. Bone structure largely normalied by 26 weeks, but the osteocyte lacunocanalicular network remained disordered. This indicates that (i) ^10kb^Dmp1-Cre-ERT2-induced recombination is not uniform through the skeleton or between sexes, and (ii) short-term tamoxifen administration has both transient and persistent effects on bone structure. Investigators using any tamoxifen-inducible system should test recombination in each site and sex, and control for tamoxifen’s effects on skeletal structure.

The Cre-ERT2/LoxP transgenic system is used for a wide range of research applications, including to study angiogenesis, muscle biology, immunology, neurobiology, and bone cell biology (1). It requires the use of tamoxifen, a synthetic selective estrogen receptor modulator, to induce gene recombination. This enables gene recombination to be induced at a chosen moment in the lifetime of the animal. Induced recombination is often targeted to restricted cell populations by using promoter regions of cell-specific genes to direct Cre recombinase expression. The system is used for testing effects of gene deletion or overexpression and, when combined with reporter genes, to study the fate of targeted cells and their progeny (lineage tracing) (2, 3, 4, 5). The temporal specificity provided by timed tamoxifen administration enables gene recombination at specific developmental or postnatal stages. This can be particularly useful in studying genes where deletion during embryogenesis is lethal or for studying the role of a gene or the fate of cells after the developmental period.

The Cre-ERT2/LoxP system makes use of transgenic expression of Cre-ERT2, a tamoxifen-sensitive fusion protein of Cre DNA recombinase with a mutant ligand-binding domain of the human estrogen receptor (ERT2) (6, 7). When tamoxifen is administered, it is oxidized in the liver to its active metabolite 4-hydroxytamoxifen (4-OHT) (3, 4). While ERT2 is resistant to endogenous estrogens, tamoxifen and 4-OHT both bind with high affinity, with 4-OHT having an affinity approximately 100 times greater than tamoxifen (5, 8). Cre-ERT2 is only active when tamoxifen exposes its nuclear translocation signal (9). This enables translocation to the nucleus, where the Cre-ERT2 complex recognizes the palindromic sequences within the two tandemly-repeated loxP sites in the target transgene, thereby excising or inverting the intervening DNA segment.

Tamoxifen also acts on endogenous estrogen receptors as a selective estrogen receptor modulator. It has antiestrogenic effects in mammary tissue (10), but mimics the inhibitory effect of estrogen on bone resorption by directly inhibiting osteoclast progenitor differentiation (11) and by increasing osteoclast apoptosis (12). Long-term tamoxifen administration protects against ovariectomy-induced bone loss by suppressing remodeling (13) resulting in high bone mass in intact mice (14, 15, 16, 17, 18), an effect associated with high levels of osteoblast and osteoclast transcripts (16, 17). These long-term effects of tamoxifen on trabecular bone mass have been documented (14, 15). However, studies of short term tamoxifen treatment, as used to induce gene recombination, have been limited to single sex studies in young mice using high tamoxifen doses, and have mainly assessed only the internal bone network (trabecular bone) or the midpoint of bone’s thick outer shell (cortical bone) (16, 17, 19, 20). Since tamoxifen also promotes longitudinal bone growth (14), it is likely to influence cortical maturation, a process by which porous, poorly mineralized bone is gradually converted into mature layered (lamellar) bone through cycles of bone remodeling (21, 22, 23). This process of cortical maturation can be observed as a spatial gradient of cortical maturation along the bone with increasing distance from the growth plate at least until the age of 26 weeks in mice (21, 22). Limiting cortical analysis to the midpoint (diaphysis) would overlook any effect of tamoxifen on this process.

While the ^10kb^Dmp1-Cre-ERT2 model is widely used to target gene recombination to mature osteoblasts and osteocytes (24, 25, 26, 27), the extent of tamoxifen-induced gene recombination in these two cell populations in trabecular and cortical bone has not been defined. Here, we tested the immediate and long-term effects of short-term tamoxifen administration in young adult mice (24) on cortical and trabecular bone structure, and on gene recombination in osteoblasts and osteocytes in the ^10kb^Dmp1-Cre-ERT2 model. While there was a persistent and high level of gene recombination in osteocytes, particularly in the cortical metaphysis in female mice, cells with recombination in trabecular bone declined dramatically with time. This administration protocol also induced a transient increase in trabecular bone volume (BV), delayed cortical consolidation, and permanently disrupted the osteocyte network. Studies using any tamoxifen-inducible Cre-ERT2/loxP system must therefore be interpreted in the light of these short- and long-term changes to the skeletal structure, and, if using the ^10kb^Dmp1-Cre-ERT2 model, should note that gene recombination is not uniform throughout the skeleton.

## Results

### Tamoxifen-induced ^10kb^Dmp1-Cre-ERT2-mediated recombination in cortical osteocytes is persistent and is greater in females and in the metaphysis

**Figure 1:**
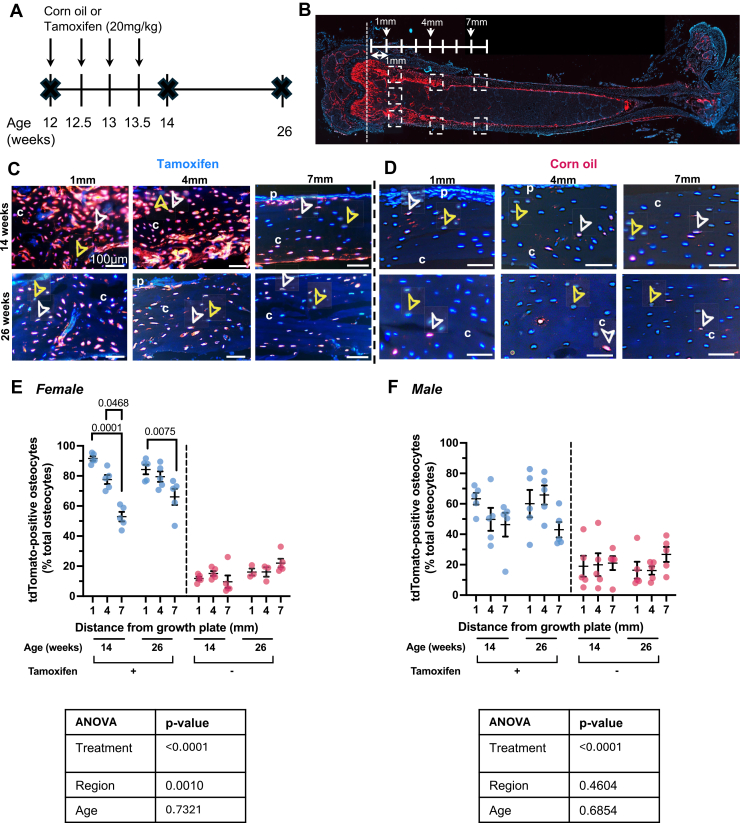
**Proportion of tdTomato fluorescent osteocytes in cortical bone in female and male *iDmpCreAi9* mice soon after and 12 weeks after tamoxifen or corn oil administration.***A*, schematic of the experiment: tamoxifen or corn oil were administered between 12 and 13.5 weeks of age, and tissues were collected prior to administration (at 12 weeks of age), at 3.5 days (at 14 weeks of age), or 12 weeks (at 26 weeks of age) after administration. All mice were *iDmpCre*^*+*^*.Ai9*. *B*, cortical and trabecular regions of measurement at 1 mm, 4 mm, and 7 mm distal to the proximal femoral growth plate. *C–F*, tdTomato-positive osteocytes in cortical bone at 1 mm, 4 mm, and 7 mm distal to the femoral proximal growth plate. Representative micrographs in 14- and 26-week-old female mice administered tamoxifen (*C*) or corn oil (*D*) *white arrowheads*: tdTomato-positive osteocytes, *yellow arrowheads*: tdTomato-negative osteocytes, p: periosteum, c: cortical bone, the scale bar represents 100 μm. tdTomato-positive osteocytes measured as a percentage of the total number of osteocytes in 14- and 26-week-old female (*E*) and male (*F*) mice administered tamoxifen or corn oil; values are mean ± SEM, n = 5 to 6 mice/group. Tables indicate *p*-values determined by two-way ANOVA, and brackets in graphs indicate *post hoc* results of Tukey’s multiple comparisons tests.

We first assessed tamoxifen-induced ^10kb^Dmp1-Cre-ERT2 (*iDmp1CreAi9*) recombination in osteocytes in cortical bone. We observed many tdTomato-positive osteocytes, and some tdTomato-positive cells on bone surfaces, including the endocortical surface and surfaces of intracortical pores in the metaphysis (Fig. 1*C*).

The proportion of tdTomato-positive osteocytes seemed greater in *iDmp1Cre.Ai9* mice at the metaphysis than the diaphysis both soon after tamoxifen administration (at 14 weeks of age) and 12 weeks later (Fig. 1*C*). Since such regional variation in ^10kb^Dmp1-Cre-ERT2 recombination has not been reported, we quantified this in osteocytes in three regions along the length of the cortex. In 14-week-old female mice, ∼80 to 90% of osteocytes in the two regions closest to the proximal growth plate (1 mm and 4 mm) were positive for tdTomato (Fig. 1, *C* and *E*). However, at the diaphysis (7 mm distal to the proximal growth plate), the percentage of tdTomato-positive osteocytes was significantly lower (∼50%) compared to the other regions. Similar results were seen 12 weeks after tamoxifen administration (at 26 weeks of age): both metaphyseal regions (1 and 4 mm proximal to the growth plate) retained a high (∼80%) level of tdTomato-positive osteocytes, and this was significantly lower (∼65%) in the diaphysis (Fig. 1*E*). This indicates that, while gene recombination was significant in the diaphysis, it was most effective in the metaphyseal cortex.

Male mice exhibited a lower proportion of tdTomato positive osteocytes and greater variability than female mice: only ∼60% of osteocytes in the 1 mm region proximal to the growth plate were tdTomato-positive at 14 and 26 weeks, and no significant difference was detected between cortical regions (Fig. 1*F*). This indicates that at both 14 and 26 weeks of age, the same tamoxifen regimen induces greater recombination in osteocytes in female mice than male mice, and with less variability, but only in the metaphysis.

Since tamoxifen-independent recombination has been reported (27, 28), we also assessed this, and found the proportion of tdTomato-positive osteocytes in corn oil-treated mice was significantly lower (∼15–20%) than in tamoxifen-treated mice at both timepoints in both sexes and at both timepoints and showed no significant variation with region or sex (Fig. 1, *C–F*).

These results indicate that short-term tamoxifen-induced recombination in osteocytes of cortical bone by ^10kb^Dmp1-Cre-ERT2 is more effective at the metaphysis and in female mice and is retained for at least 12 weeks after tamoxifen administration.

### Tamoxifen-induced ^10kb^Dmp1-Cre-ERT2-recombined cells are lost over time in trabecular bone

**Figure 2:**
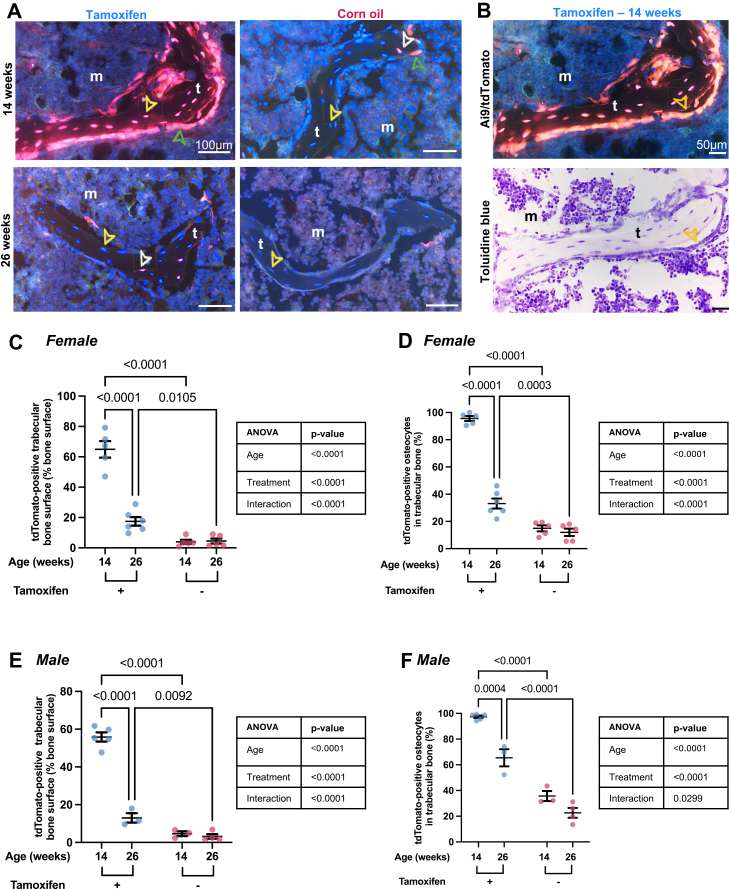
**tdTomato-positive cells on and in trabecular bone at 14 and 26 weeks of age in*iDmp1CreAi9*mice administered tamoxifen or corn oil.***A*, representative micrographs of trabecular bone in 14 and 26 week old female mice administered tamoxifen or corn oil according to the protocol shown in Figure 1*A*; *white arrowheads*: tdTomato-positive osteocytes, *yellow arrowheads*: tdTomato-negative osteocytes, *green arrowheads*: tdTomato-positive trabecular bone surface, m: bone marrow, t: trabecular bone, the scale bar represents 100 μm. *B*, serial cryosections of femora from 14-week-old female mice administered tamoxifen stained with Toluidine blue*;orange arrowhead*: tdTomato-positive osteoblasts, the scale bar represents 50 μm. *C–F*, measurements in the trabecular bone region of male and female *iDmpCre.Ai9* mice at 14 and 26 weeks of age; tdTomato-positive trabecular bone surface in female (*C*) and male (*E*) femora and tdTomato-positive osteocytes in trabecular bone of female (*D*) and male (*F*) mice administered tamoxifen and corn oil; values are mean ± SEM, n = 3 to 5 mice/group. Tables indicate *p*-values determined by two-way ANOVA, and brackets in graphs indicate *post hoc* results of Tukey’s multiple comparisons tests.

We then assessed recombination in trabecular bone. After tamoxifen injection, most of the trabecular bone surface and a large proportion of trabecular osteocytes were tdTomato-positive at 14 weeks of age (Fig. 2*A*). Toluidine blue staining confirmed that the tdTomato-positive cells on the bone surface included both cuboidal osteoblasts and flattened bone lining cells (Fig. 2*B*). By 26 weeks of age, the proportion of tdTomato-positive osteocytes and positive trabecular bone surfaces were both greatly reduced but some osteocytes, generally those located closer to the trabecular bone surface, were still tdTomato-positive.

When quantified in female mice at 14 weeks of age, tamoxifen led to recombination on ∼65% of trabecular bone surfaces (Fig. 2*C*) and in ∼100% of trabecular osteocytes (Fig. 2*D*). However, 12 weeks after the last tamoxifen injection (at 26 weeks of age), tdTomato-positive trabecular bone surface was reduced to ∼20%, and only ∼30% of trabecular osteocytes were positive.

The recombination specificity and reduction in the labeled cells in trabecular bone were very similar in male mice. Approximately 60% of trabecular surfaces were positive for tdTomato soon after tamoxifen administration (Fig. 2*E*), and this reduced to only ∼10% by 26 weeks of age (Fig. 2*E*). Close to 100% of osteocytes were tdTomato-positive in male mice at 14 weeks of age (Fig. 2*F*), as observed in female mice. This declined to ∼60% at 26 weeks (Fig. 2*F*), a lesser decline than observed in female mice.

In corn oil-treated mice, there were very few tdTomato-positive cells. In female mice, this nonspecific tamoxifen-independent recombination was ∼5% on trabecular bone surfaces and ∼10% in trabecular osteocytes and did not differ significantly with age (Fig. 2, *C* and *D*). Male mice also exhibited a low (∼5%) level of nonspecific recombination on trabecular bone surfaces (Fig. 2*E*), but the level of nonspecific recombination in trabecular osteocytes was higher in males at ∼30% at both ages (Fig. 2*F*).

These results suggest that short-term tamoxifen administration in young adult ^10kb^Dmp1-Cre-ERT2 mice induces a high level of recombination in trabecular osteoblasts, bone lining cells, and osteocytes in both male and female mice. Tracing these labeled cells and their further differentiated progeny over time (from 14 to 26 weeks of age) revealed that all these cell subsets decline dramatically with age, and the loss of recombined osteocytes in trabecular bone was greater in female mice than in male mice.

### Short term tamoxifen administration increases longitudinal bone growth while suppressing radial bone growth

**Figure 3:**
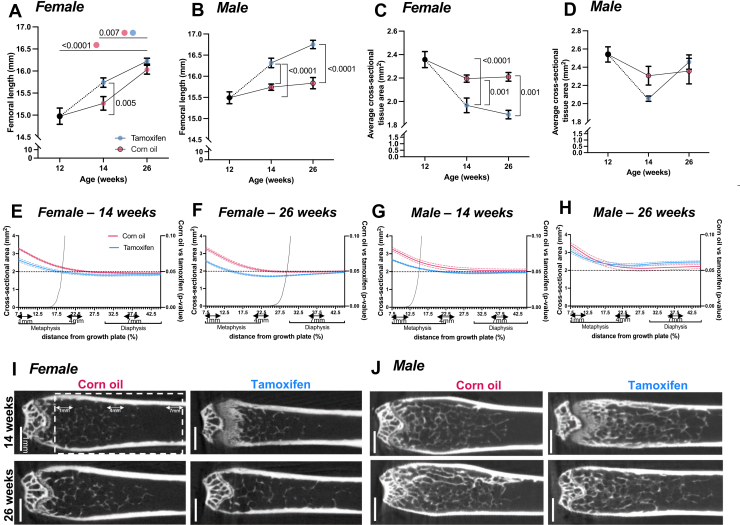
**Longitudinal and radial growth of femora from female and male *iDmpCreAi9* mice administered tamoxifen or corn oil.***A–D*, femoral length (*A* and *B*) and metaphyseal cross-sectional area (*C* and *D*) in 12-, 14- and 26-week-old female (*A* and *C*) and male (*B* and *D*) *iDmp1CreAi9* mice administered tamoxifen (*blue circles*) or corn oil (*red circles*), as described in Figure 1*A*. 12-week-old mice were untreated (*black circles*). *E–H*, femoral cross-sectional area measured every 9 μm from the metaphysis to the diaphysis in female (*E* and *F*) and male (*G* and *H*) mice administered corn oil (*red lines*) or tamoxifen (*blue lines*) at 14 weeks (*E* and *G*) and at 26 weeks (*F* and *H*); values are mean ± SEM, n = 5 to 6 mice/group, *p*-values comparing tamoxifen and corn oil at each slice determined by two-way ANOVA with Tukey’s multiple comparisons test are illustrated by the *black line*; values are shown on the right y-axis. 1 mm, 4 mm, and 7 mm regions of interest measured by cryo-histology (Figs. 1 and 2) are indicated below for comparison. *I* and *J*, representative micro-CT images of the distal metaphysis and diaphysis of female (i) and male (j) *iDmpCreAi9* mice; *dashed lines* indicate the regions of interest used for micro-CT analysis shown in *panels* (*E–H*), and 1 mm, 4 mm, and 7 mm regions indicate regions measured by cryo-histology. The scale bar represents 1 mm. micro-CT, micro-computed tomography.

While conducting micro-computed tomography (micro-CT) analysis, we noted that mice given tamoxifen had longer femora than controls. Female control mice were still growing at the time of tamoxifen administration: they demonstrated a significant, albeit moderate (<10%), increase in femoral length between 12 and 26 weeks of age (Fig. 3*A*). This was transiently accelerated by tamoxifen, indicated by a greater increase in length between 12 and 14 weeks. By 26 weeks, the bone length had normalized in the tamoxifen-treated mice. A transient acceleration in longitudinal growth was also observed in the same mice in the fourth lumbar vertebrae (L4) (Fig. S1*A*).

Male mice had longer femora than females at 12 weeks and had ceased measurable growth: male mice given corn oil exhibited no increase in femoral length between 12 and 26 weeks (Fig. 3*B*). However, tamoxifen administration transiently promoted longitudinal growth between 12 and 14 weeks of age, leading to significantly longer femurs in tamoxifen-treated males at both 14 and 26 weeks of age (Fig. 3*B*).

Femora from both male and female mice treated with tamoxifen exhibited greater growth plate width, with both the proliferating and hypertrophic zones being significantly wider (mean ± SEM proliferating zone: female control: 59.3 ± 5.8, female tamoxifen: 92.3 ± 9.9, male control: 51.2 ± 7.5, male tamoxifen: 81.7 ± 12.2, *p* = 0.0052, two-way ANOVA. Hypertrophic zone: female control: 23.1 ± 2.9, female tamoxifen: 53.1 ± 10.4, male control: 29.7 ± 9.2, male tamoxifen: 53.7 ± 4.3, *p* = 0.0059; two-way ANOVA).

We also noticed a clearly narrower metaphyseal circumference with tamoxifen administration (Fig. 3*I*). Indicated by average cross-sectional area, radial growth (the increase in cross sectional area) of female corn oil-treated mice had ceased between 14 and 26 weeks of age (Fig. 3*C*). Female mice given tamoxifen had narrower femoral metaphyses than corn oil-treated controls at both 14 and 26 weeks. However, there was no effect of tamoxifen on average cross-sectional area in males (Fig. 3*D*).

To determine whether the tamoxifen-induced reduction in average cross-sectional area in female mice was local to activity at the growth plate, we assessed cross-sectional area along the length of the femur. This confirmed that the tamoxifen-induced femoral narrowing in female mice was restricted to the metaphysis at 14 weeks of age (Fig. 3*E*) and extended a further 1 mm towards the diaphysis by 26 weeks of age (Fig. 3*F*), consistent with the 1 mm increase in femoral length during this time frame. We also measured the immediate chondro-osseous border region of the metaphysis (*i.e.* even closer to the growth plate). The femoral cross-sectional tissue area was significantly smaller in this entire region in tamoxifen-treated mice than in corn-oil treated controls at 14 weeks of age (Fig. S2*A*), but this normalized by 26 weeks of age (Fig. S2*B*), suggesting a growth-plate associated impact on radial growth that showed full recovery over time.

In male mice, although there was no effect of tamoxifen on average cross-sectional tissue area, analysis along the length of the bone at 14 weeks of age revealed a significantly smaller cross-sectional tissue localized to the proximal metaphysis approximately 1 mm from the distal growth plate (Fig. 3*G*). This too had recovered by 26 weeks of age (Fig. 3, *H* and *I*).

These results reveal sex-specific changes to skeletal structure induced by tamoxifen, including a transient induction of longitudinal growth in both male and female mice, and more extensive suppression of radial growth in female mice, particularly close to the growth plate.

### Short-term tamoxifen administration transiently increases trabecular bone mass

**Figure 4:**
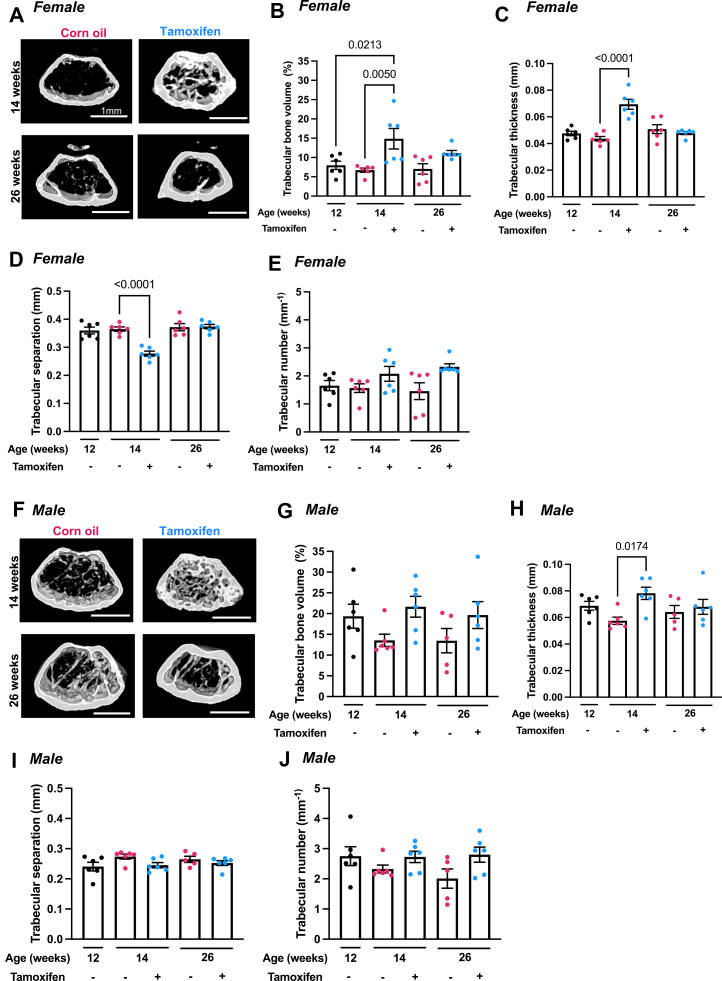
**Femoral trabecular structure in female and male *iDmpCreAi9* mice administered tamoxifen or corn oil.***A* and *F*, representative thresholded micro-CT images of the top slice of the femoral metaphyseal region in female (*A*) and male (*F*) mice showing time-dependent and treatment-dependent changes in trabecular structure; the scale bar represents 1 mm. *B–J*, trabecular bone volume (*B* and *G*), trabecular thickness (*C* and *H*), trabecular separation (*D* and *I*), and trabecular number (*E* and *J*) in female (*B–E*) and male (*G–J*) 12-, 14-, and 26-week-old *iDmpCreAi9* mice administered tamoxifen (*blue circles*) or corn oil (*red circles*) as described in Figure 1*A*. 12-week-old mice were untreated (*black circles*). Values are mean ± SEM, n = 5 to 6 mice/group, *p*-values determined by one-way ANOVA followed by Tukey’s multiple comparisons test. micro-CT, micro-computed tomography.

Next, we assessed the impact of tamoxifen on trabecular bone structure. Tamoxifen administration in female *iDmpCreAi9* mice increased trabecular bone volume (BV/TV) compared to corn oil-treated controls by 14 weeks, but this dissipated by 26 weeks (Fig. 4, *A* and *B*). The increase in BV/TV was associated with a transient increase in trabecular thickness (Fig. 4*C*), and a transient decrease in trabecular separation (Fig. 4*D*) but no change in trabecular number (Tb.N) (Fig. 4*E*). A transient tamoxifen-induced increase in trabecular bone mass was also observed at the L4 in female mice (Fig. S1).

Male mice exhibited greater variability in trabecular structure, with no significant change in femoral BV/TV, trabecular separation or Tb.N with tamoxifen administration at 14 weeks of age (Fig. 4, *F–J*) although there was a transient increase in trabecular thickness (Fig. 4*H*).

This indicates that the increase in trabecular bone mass induced by tamoxifen administration to young adult mice is strongest in female mice and is transient.

### Tamoxifen transiently increases cortical porosity at the metaphysis

**Figure 5:**
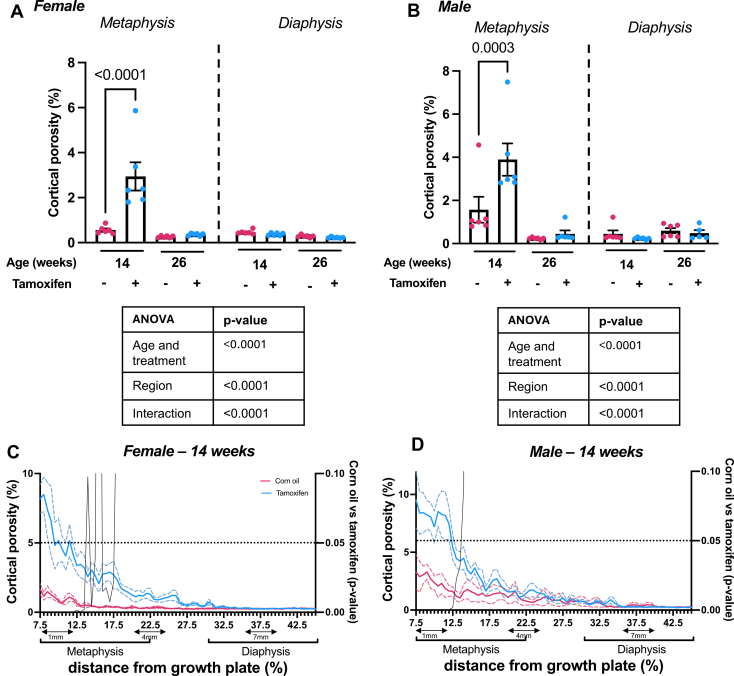
**Cortical porosity at the femoral metaphysis and diaphysis of female and male *iDmpCreAi9* mice administered tamoxifen or corn oil.***A* and *B*, femoral cortical porosity of 12-, 14-, and 26-week-old female (*A*) and male (*B*) old *iDmpCreAi9* mice administered corn oil (*red circles*) or tamoxifen (*blue circles*), as described in Figure 1*A*. Tables indicate *p*-values determined by two-way ANOVA, and brackets in graphs indicate *post hoc* results of Tukey’s multiple comparisons test. *C* and *D*, femoral cortical porosity measured every 9 μm from the metaphysis to the diaphysis in female (*C*) and male (*D*) mice at 14 weeks of age following administration of corn oil (*red lines*) or tamoxifen (*blue lines*); values are mean ± SEM, n = 5 to 6 mice/group, *p*-values comparing tamoxifen and corn oil at each slice determined by two-way ANOVA with Tukey’s multiple comparisons test are illustrated by the *black line*; values are shown on the right y-axis. 1 mm, 4 mm, and 7 mm regions of interest measured by cryo-histology (Figs. 1 and 2) are indicated below for comparison.

In micro-CT images of 14-week-old tamoxifen-treated female and male mice, the metaphyseal cortical bone surrounding the increased trabecular bone mass appeared to be poorly consolidated (Fig. 3, *I* and *J*). Indeed, metaphyseal cortical porosity in female and male mice administered tamoxifen was significantly greater than that of corn oil-treated controls at 14 weeks (Fig. 5, *A* and *B*). When analyzed along the length of the bone, tamoxifen administration in both male and female mice led to a 4 to 8 fold increase in porosity at the proximal end of the metaphysis relative to the growth plate (Fig. 5, *C* and *D*). This was not observed in the diaphysis, nor at 26 weeks of age (Fig. 5, *A* and *B*).

### Tamoxifen initially increases low- and mid-density bone content, leading to a higher proportion of high-density bone at the metaphysis

Since we observed a tamoxifen-induced increase in cortical porosity, we also assessed whether tamoxifen administration promoted accumulation of low-density bone by segregating bone areas into low-, mid-, and high-density bone (22).

**Figure 6:**
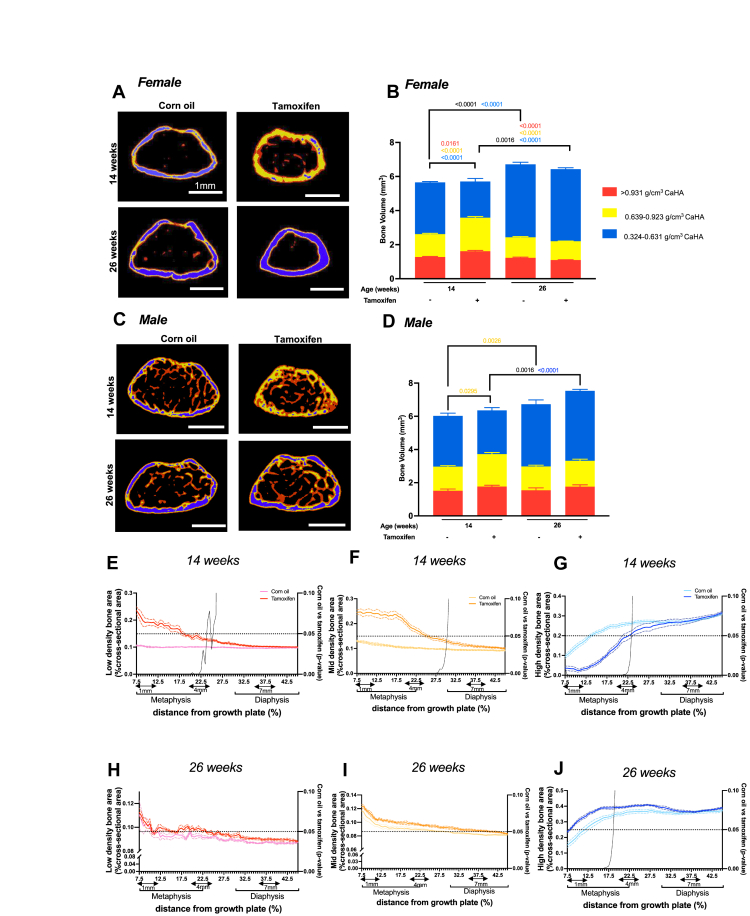
**Proportions of low-, mid-, and high-density bone in femora of female and male *iDmpCreAi9* mice administered tamoxifen or corn oil.***A* and *C*, pseudo-colored images based on multilevel thresholds of representative femora from female (*A*) and male (*B*) mice at the first slice of the metaphysis; the scale bar represents 1 mm *B* and *D*, femoral bone volume segregated into low- (*red*), mid- (*yellow*), and high-density (*blue*) volumes, at 14 and 26 weeks of age in female (*B*) and male (*D*) mice; *p*-values in *black* indicate significant changes in total bone volume, while colored *p*-values represent significant changes in low-, mid-, and high-density bone. *E–J*, femoral bone area over cross-sectional area of low-density bone (*E* and *H*), mid-density bone (*F* and *I*) and high-density bone (*G* and *J*) at 14 (*E–G*) and 26 (*H–J*) weeks of age in female mice. Values are mean ± SEM, n = 5 to 6 mice/group, *p*-values comparing tamoxifen and corn oil at each slice determined by two-way ANOVA with Tukey’s multiple comparisons test are illustrated by the *black line*; values are shown on the right y-axis.

Representative images of the top metaphyseal slice of the femora of female (Fig. 6*A*) and male (Fig. 6*C*) mice showed that high-density bone (in blue) forms the greatest proportion of bone in corn-oil injected control animals. However, with tamoxifen administration, most bone in this region at 14 weeks of age was mid-density (yellow) bone; this normalized by 26 weeks.

When quantified, female control mice had the same total BV as tamoxifen-treated mice at both 14 and 26 weeks of age (Fig. 6*B*). However, the volumes of low- and mid-density bone differed—they were each double that of corn oil-treated mice at 14 weeks but normalized by 26 weeks. This indicated that tamoxifen induced formation of low- and mid-density bone at 14 weeks of age, but that this bone matured sufficiently to reach normal amounts of each density level by 26 weeks. The response to tamoxifen was similar in male mice (Fig. 6*D*).

Since mice given tamoxifen had smaller metaphyseal cross-sectional areas (Fig. 2), we determined whether the proportions of low-, mid-, and high-density bone differed along the metaphysis to the diaphysis by calculating areas of each density as a proportion of cross-sectional area. This showed that, at 14 weeks of age, the increase in low- (Fig. 6*E*) and mid-density (Fig. 6*F*) bone induced by tamoxifen was restricted to the metaphysis. In the same region, high-density bone was significantly less than in corn oil-treated controls (Fig. 6*G*), indicating both a delay in cortical pore closure, and accumulation of low- and mid-density bone with tamoxifen administration.

By 26 weeks of age in female mice, the tamoxifen-induced increase in low- (Fig. 6*H*) and mid-density (Fig. 6*I*) bone content had recovered. By this time, the metaphysis of tamoxifen-treated mice had a greater proportion of high-density bone than corn oil-treated controls (Fig. 6*J*). This indicated maturation of the less mature cortical bone deposited in the early stages after tamoxifen administration.

Male mice also exhibited a transient tamoxifen-induced increase in low- (Fig. S3*A*) and mid-density (Fig. S3*B*) bone, and a reduction in high-density bone (Fig. S3*C*) at the metaphysis which all normalized by 26 weeks of age (Fig. S3, *D–F*).

### Cortical bone has a less-ordered osteocyte network after tamoxifen administration

**Figure 7:**
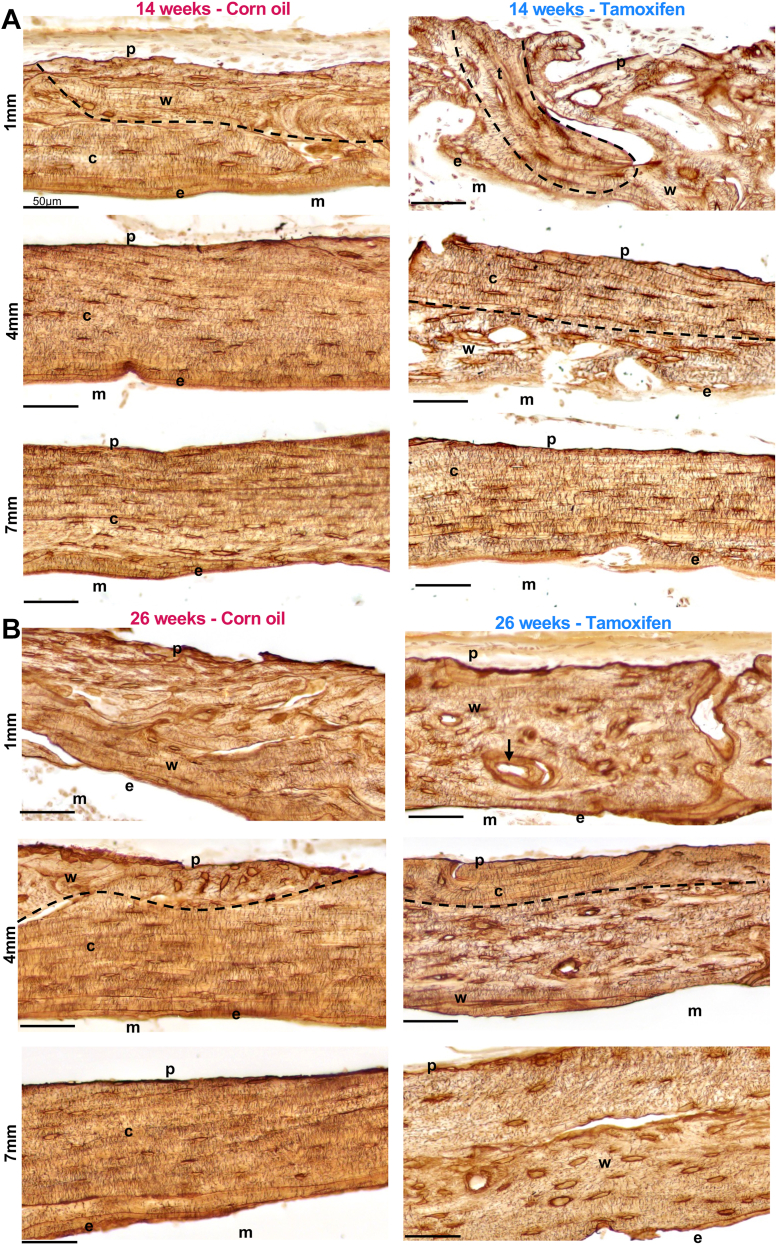
**Ploton silver stain of the osteocyte network in cortical bone of female tamoxifen and corn oil-treated controls.***A* and *B*, representative images of Ploton silver-stained cortical bone at 14 (*A*) and 26 (*B*) weeks of age, at 1 mm, 4 mm, and 7 mm distal to the growth plate. The scale bar represents 50 μm, p: periosteum, m: marrow, e: endocortical surface, c: lamellar cortical bone, w: woven bone, and t: trabecular bone. *Dashed lines* distinguish woven bone from lamellar bone, and *arrows* indicate *cement lines*.

Since an increase in cortical porosity can also be reflected in a disorganized lacunocanalicular network (22), and we assessed this by Ploton silver staining. In both 14- and 26-week-old corn oil-treated mice, the osteocytic lacunocanalicular system in the metaphyseal region (1 mm proximal to the growth plate) exhibited two distinct morphologies. Adjacent to the endocortical surface, the pattern was characteristic of osteocytes in lamellar bone, with flattened lacunae, oriented parallel to the bone surface, regular spacing, and well-ordered canaliculi, largely perpendicular to the cell bodies. On the periosteal surface, osteocyte lacunae were rounded, and their spacing, orientation, and canaliculi were less-ordered, consistent with the presence of woven bone (Fig. 7*A*). At 4 mm and 7 mm proximal to the growth plate, most osteocytes were flat and well-organized (Fig. 7*A*). This is consistent with prior observations that a mature cortical structure contains mainly lamellar bone (29), while the region closest to the growth plate contains mainly woven bone (21, 22).

With tamoxifen administration, the cortical structure at the metaphysis was extremely porous, as indicated by micro-CT (Fig. 5), and the osteocyte network within cortical bone was different to controls. At 14 weeks of age, osteocytes within the cortical region closest to the growth plate generally lacked parallel orientation and regular spacing, and their lacunae were spaced irregularly; only small regions of lamellar orientation were observed, and not consistently on the endocortical region (Fig. 7*A*) At 4 mm proximal to the growth plate (Fig. 7*A*), only cortical bone near the periosteal surface was lamellar, while cortical bone near the endocortical surface was woven and porous. At the diaphysis, the osteocyte network structure was consistent with controls and was entirely lamellar (Fig. 7*A*).

At 26 weeks, while the high cortical porosity phenotype of mice treated with tamoxifen had normalized (Fig. 5), the cortex still retained a lacunocanalicular network that reflected woven bone structure, particularly at 1 mm proximal to the growth plate (Fig. 7*B*); all osteocytes within that region had rounded lacunae, and lacked regular spacing and parallel orientation. Cement lines surrounded cortical pores, suggesting that pores present due to bone resorption at 14 weeks had been filled with bone (Fig. 7*B*), consistent with the reduction in cortical porosity at this age (Fig. 5). At 4 mm proximal to the growth plate (Fig. 7*B*), as observed at 14 weeks, only cortical bone closest to the periosteal surface was lamellar, with most of the cortex retaining a disordered osteocyte network structure. In the diaphysis (Fig. 7*B*), which contained lamellar bone, the network was still poorly connected and exhibited rounded, irregularly spaced osteocytes lacking parallel orientation to each other.

This indicated that, even though the level of mineralization and cortical porosity recovered from tamoxifen administration, the osteocyte lacunocanalicular network retained a disordered structure at least until 12 weeks after administration.

## Discussion

We report three principal findings. Firstly, short-term tamoxifen administration to ^10kb^Dmp1-Cre-ERT2 mice produces regionally heterogeneous recombination: recombination is strong and persistent in metaphyseal cortical bone (especially in female mice), but diaphyseal cortical osteocytes show less recombination. Second, while recombined trabecular osteoblasts, lining cells, and osteocytes are abundant shortly after induction, their numbers decline with time, consistent with their replacement by bone remodeling; this loss is greater in females. Third, tamoxifen produces transient changes to skeletal structure including accelerated longitudinal growth, increased trabecular bone mass, and increased metaphyseal cortical porosity. Although the structure recovers, the associated disorganization of the osteocyte network is longer-lasting. We propose that ^10kb^Dmp1-Cre-ERT2-induced gene recombination at different sites of the skeleton is influenced by tissue immaturity and region-specific effects of tamoxifen on longitudinal bone growth; both must be considered when designing experiments using tamoxifen-induced recombination systems.

Our findings of regional variation in *iDmpCre*-induced recombination and changes in recombination levels over time have not been reported previously. The first studies describing recombination in the ^10kb^Dmp1-Cre-ERT2 model showed histological images of lacZ reporter expression in cortical bone of femur and calvariae (30) or tdTomato expression in femoral trabecular bone (24), but lacked quantification or timepoints beyond 1 week after tamoxifen injection.

**Figure 8:**
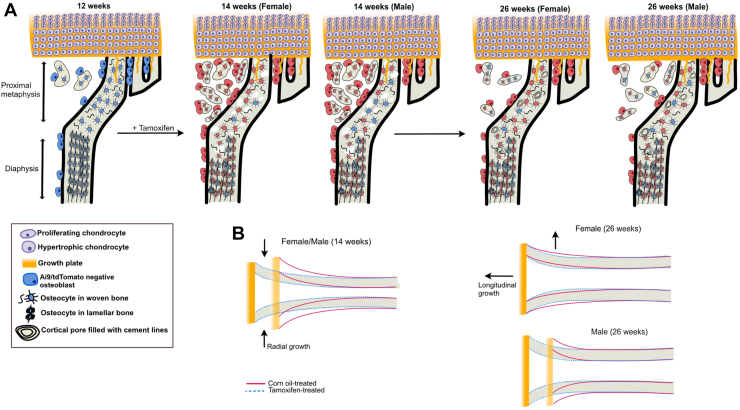
Schematic illustration of the short-term tamoxifen administration on cortical bone structure (*A*) and radial and longitudinal growth (*B*). *A*, prior to tamoxifen administration at 12 weeks of age the proximal metaphyseal cortex contains woven bone, while the diaphysis is lamellar. Initially, after tamoxifen administration (at 14 weeks), greater remodeling and woven bone formation in the proximal metaphyseal cortex causes cortical bone to become extremely porous with haphazard osteocyte arrangement. While cortical bone at the diaphysis remains lamellar, newly formed cortical bone at the endocortical (marrow-facing) surface of the metaphysis is porous and woven. Recombination in osteoblasts lining trabecular bone surfaces and osteocytes in trabecular bone are similar between males and females. Male mice have less tdTomato-positive osteocytes in the cortex at the metaphysis but not at the diaphysis compared to females. At 26 weeks, there is a partial recovery. Cortical pores are filled with bone, as evidenced by cement lines, but most of the woven bone remains unremodeled. There is a similar decline in tdTomato-positive cells lining the trabecular bone surface in both males and females, but males retain greater numbers of tdTomato-labeled osteocytes in trabecular bone compared to females. The numbers of labeled osteocytes in cortical bone remain similar to that at 14 weeks in both sexes, reflecting a lack of remodeling. *B*, between 12 and 14 weeks of age, both male and female tamoxifen-treated (*dashed lines*) femora have lengthened more than corn oil-treated controls (*solid lines*) and are narrower at the metaphyses. Between 14 and 26 weeks of age, femora of female corn oil-treated mice continue to grow longitudinally so that their femoral length catches up to that of tamoxifen-treated mice, but the femora of tamoxifen-treated mice have not expanded radially, and their cortex remains narrower. Male mice exhibit no longitudinal or radial growth during this period, and the difference in bone width and length due to tamoxifen administration observed at 14 weeks is unchanged.

The regional variation we observe here has important implications for interpreting data using the ^10kb^Dmp1-Cre-ERT2 model and other systems more recently developed to induce recombination in osteocytes (20, 31, 32). The lower level of recombination in the cortical diaphysis than the metaphysis (Fig. 8*A*) suggests that effects of ^10kb^Dmp1-Cre-ERT2 targeting on gene expression or bone structure would be more readily detected in this region than the more commonly assessed diaphysis. Changes to the metaphyseal cortex may have been overlooked since published studies to date have only assessed the cortical diaphysis (24, 26, 27).

There are several reasons why diaphyseal osteocytes may have lower levels of ^10kb^Dmp1-Cre-ERT2-induced recombination than metaphyseal osteocytes. *Dmp1* transcription, indicated by *in situ* hybridization, is greater in osteoblasts and newly-embedded osteoid-osteocytes than in deeply embedded older osteocytes (33). The metaphysis contains more early-stage osteocytes than the diaphysis because it is a region of new bone growth (21, 22). This is particularly so with tamoxifen administration, since we show here that it increases longitudinal bone growth and cortical porosity. Late osteoblasts and osteocytes that were recently embedded into the metaphyseal cortex due to growth induced by tamoxifen would therefore have higher *Dmp1* promoter activity which would facilitate greater levels of recombination. In female mice, as new trabeculae consolidated into cortical bone at the metaphysis, the higher proportion of newly-embedded osteocytes with higher *Dmp1* transcription may have driven the greater level of recombination in cortical osteocytes in female mice than males.

The proportion of tdTomato-positive osteocytes reduced with time in trabecular bone, but not in the cortex (Fig. 8*A*). At 26 weeks of age in this study, tdTomato-positive osteocytes are likely a mixture of osteocytes that were tdTomato-positive at 14 weeks that have been retained in the bone, and osteoblasts that were tdTomato-positive at 14 weeks that have become embedded in the bone matrix as osteocytes. In the trabecular region, the reduction in labeled osteocytes is likely due, at least in part, to bone remodeling, where tdTomato-positive osteocytes would be removed by bone resorption and replaced by deposition of new bone incorporating osteocytes formed after tamoxifen administration and therefore derived from progenitors without induced recombination. However, in the cortex, where there is a very low level of remodeling-associated resorption in mice (21), recombined osteocytes would be retained at a higher level.

Similarly, there was a larger decline in labeled osteocytes in female mice than in males in the trabecular compartment (Fig. 8*A*). This is likely because female mice have a higher rate of bone remodeling at sexual maturity (34, 35), and the tdTomato-positive osteocytes would have been removed and replaced. These site- and sex-specific differences in recombination (including greater variability in the cortex of male mice), cellular turnover time due to differences in remodeling, and influences of tamoxifen must be considered when using this model to study long term effects of gene recombination in the trabecular and cortical compartments.

Tamoxifen administration also led to formation of poorly consolidated bone at the metaphysis. This was indicated by higher cortical porosity, a greater proportion of low-density bone, and a more disorganized osteocyte network specific to the metaphysis (Fig. 8*A*). This formation of immature cortex was likely due to tamoxifen’s effect as a stimulus of longitudinal bone growth and inhibitor of bone resorption. A lowering in the rate of both these processes is required for cortical bone to consolidate during normal growth (22). Greater bone formation in this region was also suggested by an abundance of tdTomato-positive osteoblasts along the endocortical surface (as seen in Fig. 1*C*). Although the immature cortex consolidated over time, trabecular bone was retained in the marrow of male tamoxifen-treated mice, rather than being consolidated into cortical bone (Fig. 8*A*); this, along with their lower level of remodeling, would also explain why male mice retained more recombination in trabecular bone.

Although tamoxifen has previously been reported to increase trabecular bone mass in mice (14, 15), the long-term recovery from tamoxifen administration has not been reported previously. In an earlier study, administration to male and female mice with a 5-fold greater tamoxifen dose than the present study for four consecutive days at 4, eight or 12 weeks of age resulted in persistently high trabecular bone volume for 1 month after the last injection, but later timepoints were not tested (19). The normalization of trabecular bone mass and gross cortical structure in tamoxifen-treated mice by 26 weeks of age indicates that long-term studies of bone structure in the context of tamoxifen-dependent gene recombination would not reflect a contribution of tamoxifen. The effect of tamoxifen on bone structure is relevant for all Cre-ERT2-based recombination models that require tamoxifen-induced recombination, both for their effects on the skeleton, including studies of mineral homeostasis and metabolism, and for effects on any system that may be influenced by bone cells, including studies of angiogenesis, stem and immunological populations in the marrow, and muscle function.

Although trabecular bone mass, cortical porosity and the high proportion of low-density bone at the metaphysis normalized following tamoxifen administration, the osteocyte network did not appear to fully recover from the effects of tamoxifen by 26 weeks of age (Fig. 8*A*). Our observations are limited to unquantified histological images. The Ploton silver stain labels osteocyte lacunae, canaliculi, and cement lines (36). Since cement lines are prominent in remodeled bone (37), including in the metaphyseal bone of tamoxifen-treated mice, quantitation of the osteocyte network was not performed in this study. Further investigation would require specific osteocyte lacunocanalicular network imaging, preferably in 3D, as with Rhodamine G infiltration and confocal imaging (38). Despite this limitation, the differences we note suggest that the woven cortical bone at the metaphysis in 26-week-old tamoxifen-treated mice was not remodeled into lamellar bone as it was in controls (Fig. 8*A*). Such a continued presence of woven bone in tamoxifen-treated mice could be a consequence of a higher rate of bone remodeling (*i.e.* it is new woven bone), or a lack thereof (*i.e.* it remains from earlier disruption due to tamoxifen administration). The retention of a high level of recombination in osteocytes in cortical bone until 26 weeks, suggests the latter explanation. Unlike the transient presence of cells with tdTomato recombination in trabecular bone, all the tdTomato-positive osteocytes in cortical bone at 14 weeks are retained in the structure. This retention reflects the lack of remodeling in adult (consolidated) murine cortical bone which, once consolidated grows largely by cortical drift (21). Continued disruption of the osteocyte network, long after tamoxifen administration has ceased must be considered when assessing ^10kb^Dmp1-Cre-ERT2 mice.

The tamoxifen-induced delay in cortical consolidation was associated with a transient increase in longitudinal growth rate in both male and female mice, accompanied by metaphyseal narrowing (Fig. 8*B*). The increase in bone length and growth plate width is consistent with increased longitudinal growth rate previously reported in 10-week-old female mice administered tamoxifen daily for 4 weeks (14). This reflects its proestrogenic effects (39), and is consistent with girls, who have higher prepubertal estradiol concentrations than boys also exhibiting a higher growth velocity in this period (40).

The sex-specific effects of tamoxifen on bone length and width at 26 weeks likely reflect sex-differences in the stage of bone growth at the time of its administration: while male femora had ceased longitudinal growth by the time of tamoxifen administration, female femora were still growing. Therefore, although both female and male mice responded to tamoxifen with a transient increase in bone length, control female femora were still growing, and could therefore “catch up” with the tamoxifen-treated mice. Female tamoxifen-treated mice had narrower metaphyses than controls at both 14 and 26 weeks of age and males exhibited this only at 14 weeks of age (Fig. 8*B*). Strictly speaking, the bones are not becoming narrower, but are forming a new, narrower region through tamoxifen-induced longitudinal growth. The lengthened cartilage template formed by chondrocyte proliferation and cartilage matrix production is transformed into mineralized cartilage and bone at the metaphysis (21). By 26 weeks of age, while tamoxifen-treated female mice continue to have narrower metaphyses, male mice recover (Fig. 8*B*). This could be because in female mice, the additional strength needed due to the rapid increase in length is met by consolidation of trabeculae along the endocortical surface. In male mice, the increase in length is compensated for by a testosterone-induced increase in cross-sectional area at the periosteum.

While we used the ^10kb^Dmp1-Cre-ERT2 model in this study, other Cre-ERT2 models targeting bone and other tissues may be affected by regional differences in gene recombination. This is particularly likely in other models that target bone-embedded osteocytes, such as the *Sost*-Cre-ERT2 (31, 32) model. It may also occur in models that induce recombination in osteocytes following targeted deletion earlier in the osteoblast lineage, such as the *Osx*-Cre-ERT2 model, which targets osteoblast lineage cells from the progenitor stage onwards (41), and *Col1a1*-Cre-ERT2 (41, 42) and *Bglap/Ocn*-Cre-ERT2 (43) models, which target mature, matrix-producing and matrix-mineralizing osteoblasts, respectively. The *Ctsk*-Cre-ERT2 (44) model is used to target osteoclasts, as well as a specific population of periosteal SSPCs. Whether any of these other tamoxifen-induced recombination models exhibit regional differences in recombination has not been specifically tested. Each will need to be tested on a case-by-case basis. In addition to specific targeting of bone cells, given that osteoblasts and osteocytes have local and systemic effects on differentiation of other cell types, including endothelial cells (45), hematopoietic stem cells (46) and myoblasts (47), other tamoxifen-induced recombination systems used for studies of hemopoiesis, angiogenesis and muscle biology, should also consider short and long-term changes to skeletal structure in their models. Again, this remains to be tested.

In summary, short-term tamoxifen administration in ^10kb^Dmp1-Cre-ERT2 mice induces effective long-term recombination in osteoblasts and osteocytes, but the proportion of recombined cells differs across time, between sexes, and in different regions of bone. Tamoxifen also exerts both transient and nontransient effects on skeletal structure; while the tamoxifen-induced increases in cortical porosity and bone mass are temporary, a defective osteocyte network is retained. We conclude that studies using Cre-ERT2 models, including in adult mice, must consider tamoxifen-induced changes in bone structure, including regional variation, sex-dependency, and the loss of recombined osteocytes in trabecular bone over time.

## Experimental procedures

### Mice

*iDmp1Cre.Ai9* mice expressing tdTomato fluorescence under the control of ^10kb^Dmp1-Cre-ERT2 were generated by crossing: *iDmp1Cre* (^10kb^Dmp1-Cre-ERT2) mice (30), obtained from Alexander Robling (Indiana University), with *Ai9* (Gt(ROSA)26Sor^tm9(CAG-tdTomato)Hze^) mice (48), obtained from Anna King (University of Tasmania). Both were maintained in a C57BL/6 background.

Both male and female mice were used with *iDmp1Cre*^*+*^ littermates as controls; all samples were collected with nonidentifiable codes allocated sequentially, and therefore were random according to sex, genotype, and treatment; all analyses were performed with observers blinded to the sex, genotype, and treatment of the animals.

Ai9 recombination was induced by intraperitoneal injection of tamoxifen (Sigma-Aldrich) at 20 mg/kg dissolved in corn oil, at 12, 12.5, 13, and 13.5 weeks of age as previously established (24) (Fig. 1*A*). Controls were administered an equivalent volume of sterile corn oil (vehicle) at the same timepoints. Samples for histomorphometry and micro-CT were collected at 12, 14, and 26 weeks of age.

All animal procedures were conducted with approval of the St Vincent’s Health Melbourne Animal Ethics Committee.

### Cryo-histology and lineage tracing

Femora from 14- and 26-week-old *iDmp1Cre.Ai9* mice were decalcified and embedded in optimal cutting temperature embedding medium (49). Thin frozen longitudinal, coronal sections were cut at 7 μm and stained for Hoechst and Ai9-positive and -negative osteocytes, and positive and negative bone surfaces (for osteoblasts) were counted using the Osteomeasure system (Osteometrics) in the femoral distal metaphysis. Trabecular bone was measured within a 1 mm long area commencing 1 mm proximal to the growth plate, avoiding the primary spongiosa. Since Ai9 labeled both cuboidal and flattened bone-lining cells along the trabecular bone surface, Ai9-positive trabecular bone surface was measured as a percentage of total trabecular bone surface. Cortical osteocytes were counted on both medial and lateral sides of the femora in a 1 mm long region at 1 mm, 4 mm, and 7 mm proximal to the growth plate (Fig. 1*B*). Femoral distal growth plate widths, separated into proliferating and hypertrophic zones, were measured using Osteomeasure (Osteometrics). Selected sections were stained with Ploton silver stain and toluidine blue stain, as previously described (22, 50).

### micro-CT

Micro-CT was performed on femoral and vertebral specimens using a SkyScan 1276 system (Bruker micro-CT). Images were acquired using the following settings: 9 μm voxel resolution, 0.25 mm aluminum filter, 57 kV voltage and 200 μA current, 520 ms exposure time, 0.4 degrees rotation, frame averaging=2 and 70% partial width. Images were reconstructed and analyzed using SkyScan software: Nrecon (version 1.7.1.0), DataViewer (version 1.5.4.0), and CTAnalyser (version 1.16.4.1). For trabecular bone analysis, the femoral distal growth plate was identified, and measurements were taken in a region of interest commencing at 7.5% of the total femur length from the growth plate towards the femoral midshaft and extending for 15% of the total femur length (51). For cortical bone, the region of interest commenced at 30% of total femoral length from the growth plate towards the midshaft and extended for a further 15% of the total femur length. Slice by slice analysis are expressed as a rolling average for every 5% of distance measured (52). For trabecular analysis in the L4, the region of interest commenced at 25% of the vertebral length above the midpoint of the vertebral body and extending for a further 50% of the total vertebral length (51). Bone was defined by automatic thresholding from each dataset in CTAnalyser and manually adjusted by comparing the binary selection to the raw images. To measure low-, medium- and high-density bone, multilevel thresholding was performed using CTAnalyser as previously described; thresholds were defined based on 14-week-old control females treated with corn oil (22, 53, 54). Thresholds were as follows: trabecular and cortical bone >0.25 g/cm^3^; cortical pores <0.5 g/cm^3^, low-density 0.32 to 0.63 g/cm^3^, mid-density 0.64 to 0.92 g/cm^3^ and high-density >0.93 g/cm^3^.

### Statistical analyses

Statistical analyses were carried out using GraphPad Prism 10. One-way ANOVA, two-way ANOVA or three-way ANOVA was used, as indicated in figure legends, with Tukey’s multiple comparisons test to identify significant differences. A *p* < 0.05 was considered significant. No outliers were excluded from any analyses.

## Data availability

All data described are contained within the article or supporting information; raw data are available on request.

## Supporting information

This article contains supporting information.

## Conflict of interest

The authors declare that they have no conflicts of interest with the contents of this article.
