# Supplementary material for: Tamoxifen induces region-specific osteocytic recombination and transiently alters bone structure in Dmp1-Cre-ERT2 mice
Source: J Biol Chem. 2026 Jun 22;302(8):113275. doi: 10.1016/j.jbc.2026.113275 (PMC13400240; doi:10.1016/j.jbc.2026.113275)

Supporting Information: Figure Legends

Supporting Information Figure 1. Vertebral length and bone mass of tamoxifen-treated female *iDmp1CreAi9* mice compared to controls. a-d: Measurements at the L4 vertebrae, including L4 vertebral length (a), trabecular bone volume (b), trabecular thickness (c) and trabecular number (d) of 14 and 26-week-old female mice treated with tamoxifen or corn oil as described in Figure 1a; values are mean ± SEM, n=6 mice/group, p-values determined by one-way ANOVA with Tukey's multiple comparisons test.

Supporting Information Figure 2. Cross-sectional area in the femoral proximal metaphysis, close to the growth plate in female *iDmp1CreAi9* mice compared to corn oil-treated controls. a: Longitudinal micro-CT image of a femur from a 14-week-old female mouse (corn oil treated), showing the region measured in dashed lines directly beneath the growth plate; this is the same image shown in Figure 3i, used here for illustrative purposes. b-c: Femoral cross-sectional tissue area measured for every for every 9μm within the indicated ROI in female mice at 14 (b) and 26 (c) weeks of age after administration of corn oil (red lines) or tamoxifen (blue lines), as shown in Figure 1a; comparison between tamoxifen and corn oil are illustrated by a black line corresponding to the p-value at each slice; values are shown on the right y-axis and were determined by two-way ANOVA with Tukey's multiple comparisons test; values are mean ± SEM, n=6 mice/group.

Supporting Information Figure 3. Proportions of low-, mid- and high-density bone in femora of male *iDmp1CreAi9* mice administered tamoxifen or corn oil. a-f: Femoral bone area over cross-sectional area of low-density bone (a,b), mid-density bone (c,d) and high-density bone (e,f) at 14 (a,c,e) and 26 (b,d,f) weeks of age in male mice. Values are mean ± SEM, n=5-6 mice/group, p-values comparing tamoxifen and corn oil at each slice determined by two-way ANOVA with Tukey's multiple comparisons test are illustrated by the black line; values are shown on the right y-axis.

Supporting Information Figure 1

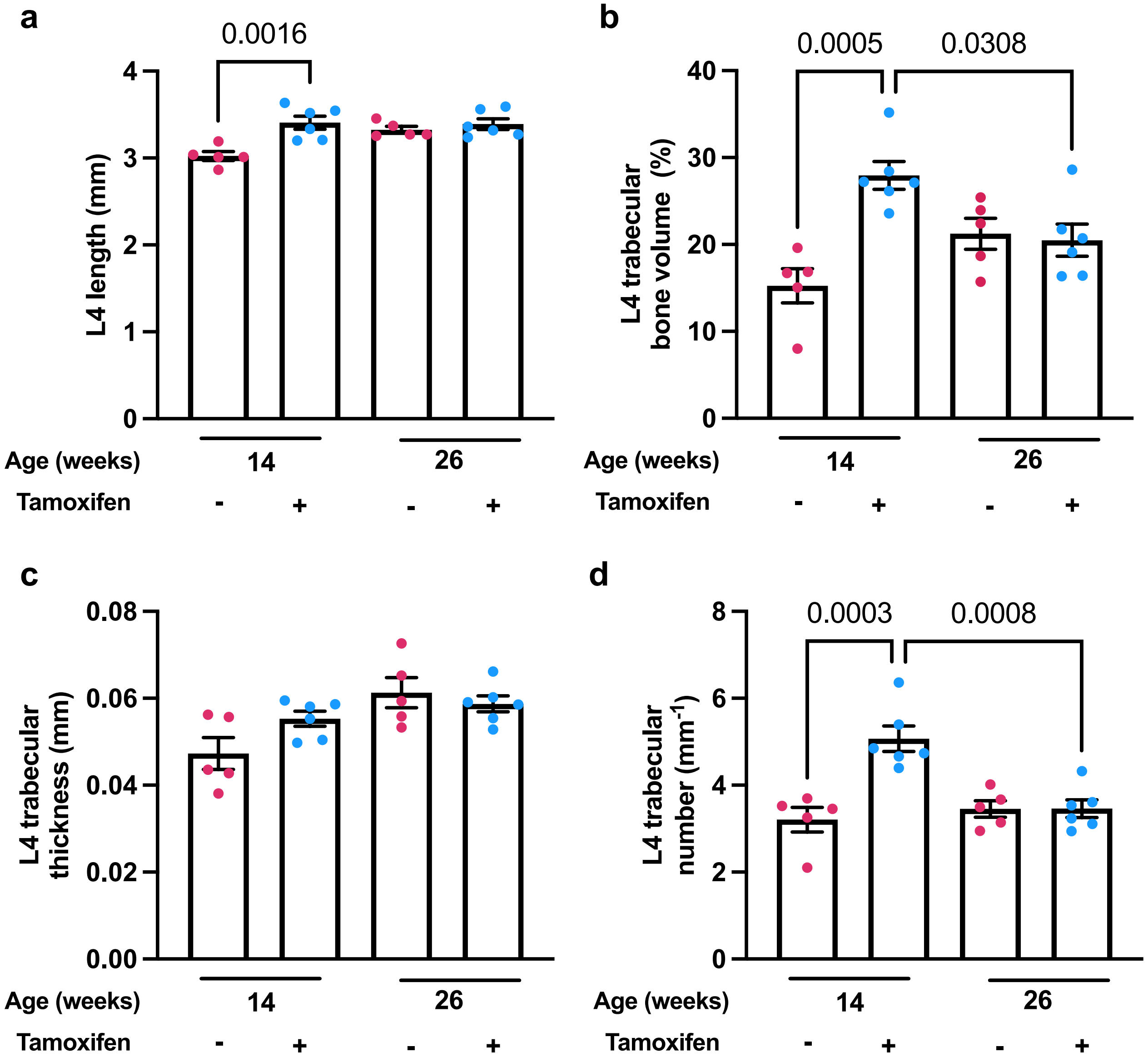

Supporting Information Figure 2

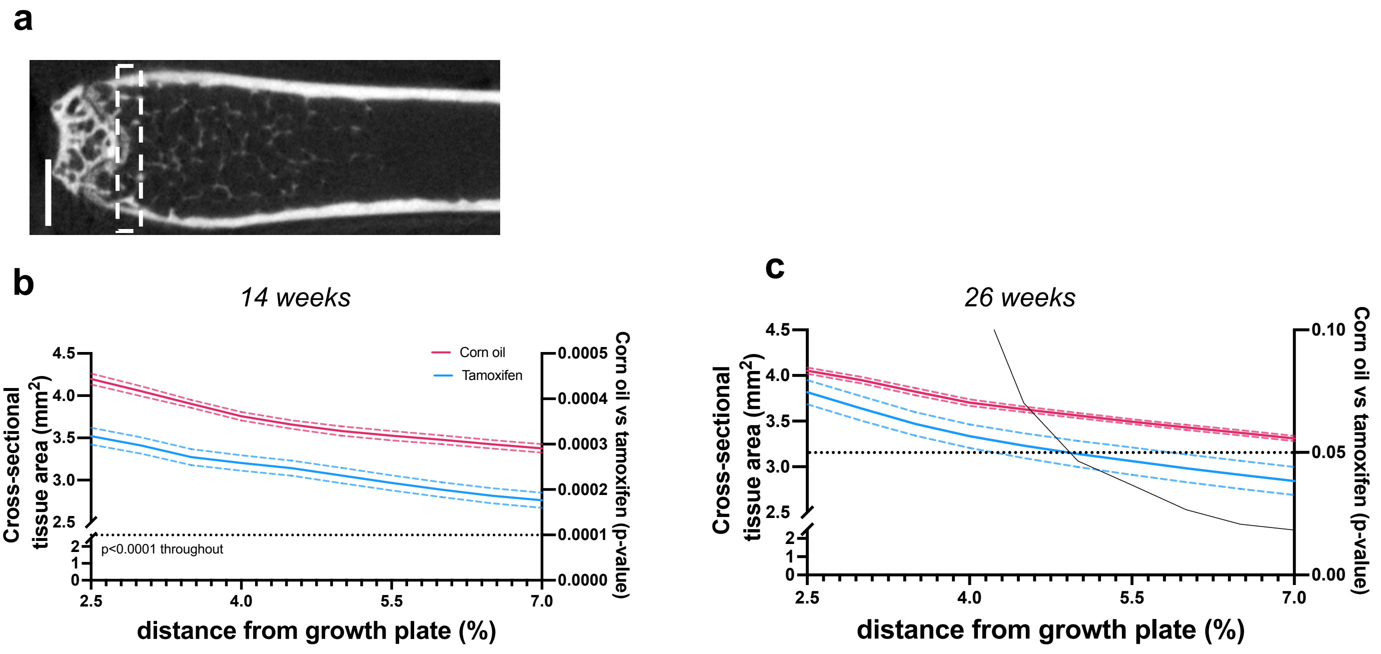

Supporting Information Figure 3

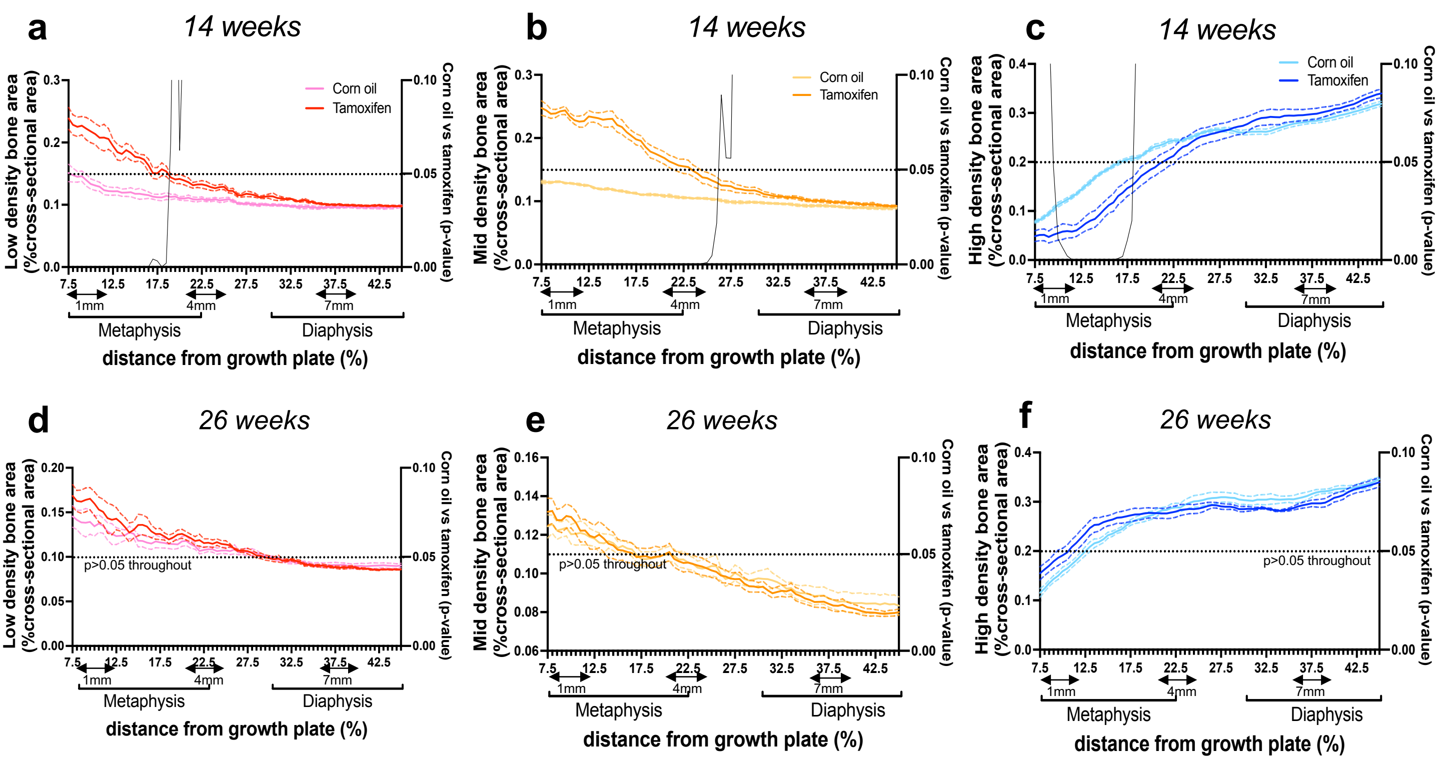

Supplement: Supporting Information [file mmc1.pdf]
